# Correlated evolution between colour conspicuousness and drum speed in woodpeckers

**DOI:** 10.1098/rsos.221096

**Published:** 2022-10-26

**Authors:** Ghislaine Cárdenas-Posada, Matthew J. Fuxjager

**Affiliations:** ^1^ Department of Ecology, Evolution and Organismal Biology, Brown University, Providence, RI 02912, USA; ^2^ Department of Biology, Wake Forest University, Winston-Salem, NC 27109, USA

**Keywords:** plumage coloration, drumming, correlated evolution, woodpeckers

## Abstract

Sexual selection drives the evolution of many spectacular animal displays that we see in nature. Yet, how selection combines and elaborates different signal traits remains unclear. Here, we investigate this issue by testing for correlated evolution between head plumage colour and drumming behaviour in woodpeckers. These signals function in the context of mate choice and male–male competition, and they may appear to a receiver as a single multimodal display. We test for such correlations in males of 132 species using phylogenetic linear models, while considering the effect of habitat. We find that the plumage chromatic contrast is positively correlated with the speed of the drum, supporting the idea that species evolving more conspicuous plumage on their head also evolve faster drum displays. By contrast, we do not find evidence of correlated evolution between drum speed and head colour diversity, size of the head's red patch, or extent of the plumage achromatic contrast. Drum length was not correlated with any of the plumage coloration metrics. Lastly, we find no evidence that habitat acts as a strong selective force driving the evolution of head coloration or drumming elaboration. Coevolution between different signal modalities is therefore complex, and probably depends on the display components in question.

## 1. Introduction

Sexual selection is responsible for producing some of the most spectacular traits in the animal world [1,2]. Iconic examples include the elaborate dance behaviour that birds of paradise use to court mates [3], or the flashy red dewlaps that *Anolis* lizards extend to fight off rival males during territorial interactions [4]. Yet, sexual selection for these traits seldom proceeds unencumbered. Forces of constraint that emerge from a species' ecology [5], physiology [6] and/or phylogenetic history [7] can act to either halt sexual selection or prevent it from driving trait evolution to extremes. Thus, a longstanding goal of evolutionary biology is to understand how sexual selection interacts with complex constraint landscapes to give rise to the diversity of incredible traits that we see across our planet.

One of the clearest illustrations of how sexual selection interacts with constraint comes from work on multimodal displays, which are displays that stimulate two or more sensory systems in a receiver [8]. This behaviour often appears as a conundrum, because it involves broadcasting several signals that should each incur their own set of costs (e.g. time, energy, etc.). The ‘transfer hypothesis' [9] (a.k.a., transference hypothesis) is a classic idea first developed by Charles Darwin to explain this phenomenon [1] and then revisited by Gilliard [9] while studying Bowerbirds. Gilliard noticed that species differed markedly with respect to the complexity of the bowers they created, such that taxa that made more complex bowers had less elaborate plumage (and vice versa). Comparative work later verified the strong negative relationship between these two variables across several bowerbird species [10], with the explanation that bower complexity probably evolves to replace plumage complexity since the former is less costly than the latter [10,11]. This principle forms the crux of the transference hypothesis: when signal modalities serve the same function, less costly signals will evolve to replace costlier ones. As a result, we sometimes see signatures of an evolutionary trade-off between different signal modalities across large groups of taxa [12]. Support for this relationship is found among species in several avian clades, such as finches, dabbling ducks and antwrens, all of which show a clear negative relationship between measures of display complexity for acoustic and visual signals [13–16].

Transference, however, is not the only model for how complex multimodal signals should (or should not) evolve. The emergence and interaction of different signal modalities can depend on a host of factors, including resource availability [17], costs [18], habitat [5] and information content [19]. For example, information content theory [19,20] suggests that animals can use multiple signals to back up one single message (e.g. one quality) to increase the accuracy and reliability of the information. In birds of paradise, this phenomenon is observed through positively correlated evolution among song complexity, plumage colour complexity and behavioural display complexity [21]. Likewise, many species use multiple signals to communicate several different messages (multiple qualities), which has an effect of increasing the net transmission of information content. Individuals, for instance, might use one signal modality to advertise to its competitors, while using a second signal modality to mediate mate choice [22]. Of course, an entirely different possibility is that different signal modalities evolve independently of each other in response to entirely different forces of selection. Characteristics of a species' habitat (e.g. vegetation density, light environment, etc.) can induce such an effect by influencing the efficacy of auditory and visual signals in a manner that impacts signalling strategy [23–25]. Indeed, this idea of sensory drive proposes that selection favours the elaboration/exaggeration of signalling traits that enhance their transmission through the environment [5,26], and it is often evoked to potentially explain the lack of any evolutionary correlation between song and plumage [27,28].

**Figure 1.**
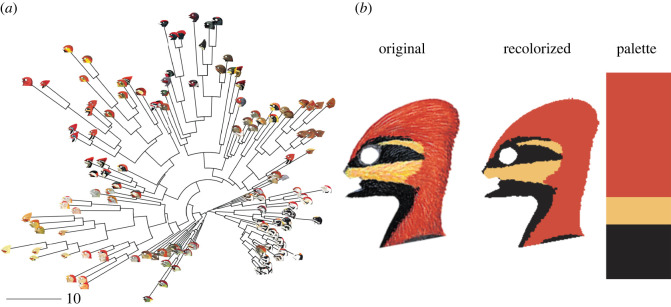
(*a*) Neighbour-joining tree representing the variation of the head plumage coloration of male woodpeckers obtained by calculating colour distances between each pair of images. Note that this tree does not depict the phylogenetic relationship among species, but rather shows how species clump together as a function of colour quantification through our analyses (see electronic supplementary material, methods). Units are Euclidean distance in CIE Lab colour space (a.k.a. units of ‘ΔE') with a range between 0 and 256, where 256 reflects the most different. (*b*) Colour segmentation performed using the ‘recolorize' package. A PNG with all non-plumage components masked using transparency (original) is loaded into the package and run through the ‘recolorize2' function, which bins all pixels into a fixed set of colour bins and then combines any colour clusters which are less than 55 ΔE apart in CIE Lab colour space, resulting in a discrete set of distinct colour patches (recolorized), along with a colour palette (palette). Use of modified illustrations was granted with permission by ©Lynx Edicions.

Here, we investigate these ideas and test how signal elaboration influences the relationship between two signalling traits in the multimodal display phenotype of woodpeckers: non-vocal drumming behaviour and conspicuous head plumage. Drumming occurs when an individual hammers its bill on a substrate to produce a loud mechanical jackhammering sound, which nearly all extant woodpecker taxa perform to help navigate territorial competition during the breeding season [29,30]. Head colour is similarly iconic in woodpeckers, with many species showing gaudy red plumage ornaments on the nape, crown, forehead and/or malar. This signal is thought to help mediate intraspecific aggression [31], but may also be involved in mate choice [32]. Both drumming and head colour are probably costly signals. Drumming, for example, may incur significant energetic costs [33], it may cost individuals in foraging time [34] or make them more conspicuous to predators [35]. These costs also probably apply to head colour, since red pigmentation in most birds (except some families, like parrots or hummingbirds) derives from carotenoids acquired through the diet [36] and conspicuous colour patches could make individuals more noticeable to predators in the environment [37,38]. Furthermore, drumming behaviour and head colour vary markedly within the woodpecker clade. With respect to the drum, species show differences in speed (beats s^−1^), length (total beats) and rhythm (Δspeed s^−1^) [39,40]. Head colour similarly varies, such that some woodpeckers have no red on their head, whereas others maintain heads that are either entirely red or that are a plethora of rich and vibrant carotenoid-base colours (reds, oranges, yellows; figure 1*a*).

We run our analysis by assessing the relationship between components of drum display and head colour across the woodpecker clade. For the drum, we focus on speed and length. Past work suggests that both can evolve under sexual selection, with faster and longer drums benefiting individuals in competitive bouts for reproductive territories [41,42]. Moreover, signatures of strong sexual selection, such as size dimorphism, positively predict species differences in drum length, but not drum speed [40]. For head colour, we focus on measures of: carotenoid coloration such as the red area size, conspicuousness such as the chromatic and achromatic contrast (white and black contrast) and colour diversity or proportion of different colour patches. Past studies suggests that some of these measures, like the size of the red area, are associated with male success at acquiring mates [43], with other work suggesting that head colour is positively related to territory quality [32] and dominance [44]. Thus, we use phylogenetically informed models to test whether these two communication signals evolve in a correlated or uncorrelated fashion, while also considering the effect of potentially important covariates, such as habitat, to test whether a generalized evolutionary pattern of display elaboration exists within the woodpecker family.

## 2. Material and methods

Drumming and plumage coloration data were collected from 132 species of woodpeckers. For drumming behaviour, we used data previously published by Miles *et al*. [39,40]. Briefly, the data used were collected from citizen scientists' acoustic recordings, which are archived in XenoCanto and the Macaulay Library of Natural Sounds (Cornell University; http://macaulaylibrary.org). Species were selected based on the availability and quality of the recording, such that only species with at least three high-quality recordings were included. From these, averages for drum speed and length were obtained. Drum speed was calculated by dividing the total number of beats in a drum by the display's total duration (s), whereas drum length was simply calculated by counting the total number of beats in the drum. Given the lack of information regarding differences in drumming between sexes, we only included in the analyses drumming data from recordings that specifically came from males [40].

### 2.1. Plumage colour data

We collected colour data from digital images of woodpecker colour plates from the *Handbook of the Birds of the World* [45]*.* Previous studies have demonstrated that assessing colour from digital images or colour plates is comparable to colour measures from the plumage of museum specimens [46–48]. This past verification gives us greater confidence that we are accurately measuring relative differences in woodpecker head colour across the family. Note that the order Piciformes (to which woodpeckers belong) probably do not have ultraviolet-sensitive opsins [49]; therefore, we expect that patches reflecting UV (if they exist) would have little value toward communication. The lack of UV reflectance data in our analyses should therefore not have an appreciable impact on our results and interpretations.

To address how a colour visual signal relates to an acoustic signal, we focused our measure of colour to feathers, avoiding colour collected from the skin or eyes. There are several reasons for this decision. First, skin colour and eye colour account for very little colour ornamental in woodpecker species (less than 5% of all taxa). Second, it is challenging to consider ‘costs' of colour between feathers and skin, since colour in these tissues are derived from different mechanisms [50].

Equally important is that we focus specifically on the head of male woodpeckers. Most taxa in the woodpecker family do not show any evidence of plumage dimorphism on the body [45]. Rather, dimorphism in these birds is largely relegated to head plumage, particularly with respect to the size, shape and diversity of red patches (figure 1*a*). Moreover, this specific trait has been linked to sexual selection [51].

To measure the head plumage colour of male woodpeckers, we imported species images in Adobe Photoshop (Adobe Inc, San Jose, CA, USA) at 300 dpi and manually removed all the bare parts (e.g. beak, eye, legs) from each image (we wanted to measure plumage coloration only, see above). We cropped each image to a file containing the head, nape and upper throat, allowing us to exclusively focus on the head. We then rotated all images to the same orientation and exported the image as a .png file with a transparent background. To ensure we were sampling evenly across all images independently of the bird/image size, we sampled each image at the maximum sampling frequency for the smallest image (e.g. width in pixels of the smallest image). This ensured we were not sampling larger images at a higher frequency, which could have biased the results. To test whether our chosen sampling frequency affected our results, we also ran these analyses using a variety of different sampling schemes, such as image-specific sampling frequencies and fixed sampling frequencies lower than the maximum frequency of the smallest image. We found that varying the sampling schemes in this range made no difference in the metrics we used in this study (slope of 1 and *r*^2^ > 0.999 for metric values across sampling schemes).

To measure plumage coloration, we first created colour maps (image segmentation maps based on colour patches) of each processed image using the *recolorize* package in R (https://github.com/hiweller/recolorize) (figure 1*b*) [52]. This approach provides a variety of tools for automatic and semi-manual colour-based image segmentation. One major advantage of *recolorize* is that it does not require users to specify the expected number of colour patches for each image, which can otherwise pose problems of repeatability and user bias for comparative analyses involving a wide range of colour patterns with non-analogous elements [53]. To generate colour maps for all of the images in our dataset, we first used the ‘recolorize()’ function to generate colour histograms of each image in standard red-green-blue (sRGB) colour space, with three bins per colour channel (resulting in 3^3^ = 27 bins for each image). This produces an intermediate colour map with a maximum of 27 colours, each representing the average colour of all the pixels in each colour bin. We then used the ‘recluster()’ function (also in *recolorize*) to combine colour patches that were highly similar in colour, using a CIE Lab distance cutoff of 45 and a D65 reference white, meaning the maximum distance between two colours is approximately 256. The resulting colour maps created by *recolorize* provided image-specific colour patches, which we assessed manually for accuracy (figure 1*b*). These colour maps were then supplied in subsequent analyses to assess several colour metrics that are standard in colour analysis studies [54].

The first colour measurement obtained was the chromatic boundary strength of adjacent colour patches (chromatic contrast) [54], which reflects how colourful the head is. Chromatic contrast is optimal for quantifying signal efficacy since it reflects an animal's conspicuousness against its background, and it is often sexually selected because it facilitates conspecific assessment [5,54]. Next, we measured the achromatic contrast, which reflects the amount of black and white contrast in the woodpecker's head. Third, we measured the head colour diversity, as computed by Simpson's diversity index [54]. This metric reflects size and diversity of colour clusters, as well as the relative difference in colour pattern among species. Lastly, we measured the size of the red coloration patch on the head, which may reflect levels of carotenoid intake and/or processing [55]. This trait is probably related to mating success and male quality in birds [32,43].

To measure the chromatic and achromatic boundary strength and the Simpson's diversity index for colour, we used boundary strength analyses of the colour-mapped images using the *adjacent* function in the *pavo* package in R [56]. Boundary strength is a measure of the contrast produced by colour pattern geometry. Adjacent colour patches that have a high degree of chromatic contrast (such as blue and red) will have higher boundary strength values, indicating a more visually stimulating colour pattern [56]. For each set of colour clusters, we provided the colour distances in CIE Lab space, so that the distances in the *a* and *b* (green-red and blue-yellow) channels were used to measure the chromatic contrast. This function returns a variety of colour pattern geometry metrics, of which we retained the mean boundary strengths and scaled Simpson's diversity index. Simpson's relative colour diversity index measures how well represented each colour is in the colour pattern; a value of 1 indicates that each colour patch is equal in size, while a value near 0 indicates that one colour patch dominates the colour pattern [54,57,58]. To measure red head colour, we used the *colordistance* package in R [59] to calculate the proportion or extension of head plumage that fell within a range of cutoffs in CIE Lab space that we determined by visual inspection: L (luminance) > 55, *a* (red-green) > 30 and *b* (blue-yellow) > 5. Higher values for all metrics generally indicate greater colour elaboration and more salient visual signal. Because all the metrics for plumage coloration could covary, we tested for multicollinearity among the variables using the *stats* and the *corrplot* packages in R [60]. We found that the covariation between variables was low (as reviewed by Taylor [61]) (less than 0.7, electronic supplementary material, table S1), therefore we did not recur to variable reduction methods.

### 2.2. Habitat data

Environment and/or habitat can influence multimodal signal evolution [5,23,24,26]; thus, to examine such effects in the context of woodpecker drum and colour evolution, we collected data for each species' primary habitat, as indicated by Stotz *et al.* [62] and Del Hoyo *et al.* [45]. We then classified these habitats in one of two broad categories—closed or open—which are thought to represent two extremes of the habitat continuum to which signalling strategies (visual and acoustic) are adapted [23,24]. We considered species inhabiting forests and woodland as living in ‘closed' environments, whereas we considered species inhabiting deserts, grasslands, etc., as living in ‘open’ environments. This approach follows previously published work that similarly assesses effects of habitats on signal evolution [28,63]. To test whether habitat relates to the elaboration of head plumage coloration or drumming behaviour, we ran phylogenetic generalized least square (PGLS) regressions, with habitat as a covariate of each model (see below).

### 2.3. Comparative analysis

We used a phylogenetic comparative approach to test how acoustic and visual signalling modalities potentially coevolve across woodpecker species. Non-phylogenetic analyses assume independence among data points, but this assumption can be violated by ancestral relatedness among taxa in comparative studies [64]. Thus, we accounted for the shared history of woodpeckers through analyses informed by recently published maximum clade credibility supermatrix tree (based on nuclear and mitochondrial genes), which is time-calibrated to fossil and biogeographic data [65]. We then added a time calibration and taxon name adjustment based on previous work [66]. This tree had strong bootstrap support (greater than 90) for branches leading to the major taxonomic groups included in the present study and has previously been used in several studies with great confidence [39,40,67,68]. Analyses were run in R (v. 3.6.3) [69] using a significance level of *p* < 0.05.

We tested for correlated evolution between drumming and colour in woodpeckers using PGLS regression models, which is the standard approach to addressing our question of interest [13,28]. PGLS analyses were run using the package ‘caper' [70], where we allowed the phylogenetic signal in the residuals (*λ*) to be optimized towards its maximum-likelihood value [71]. Our models used plumage coloration traits as predictors of the behavioural traits (drumming), as behavioural traits are more labile and could be more responsive to selection. However we also run the models using the drumming traits as predictors of the colour traits to ensure our results were robust (see electronic supplementary material) [13,27,72]. To conform to parametric norms, we log_10_ transformed all the variables before running the models. For all models, we used habitat as a covariate to test for the effect of this ecological factor on the relationship between the signalling traits.

Previous work reports a negative relationship between body size and drum speed [40]; thus, to account for this relationship, we followed a procedure described by Garland *et al.* [73] and ran a linear regression between drum speed and species' average body mass. Then we extracted the residuals from this analysis and used them as measures of relative speed for PGLS analyses. Additionally, other studies have found that the evolution of plumage colour, particularly carotenoid-based plumage, depends on the body size [74,75]; therefore, we looked at any associations between these two metrics using the *corrplot* package, but we found no significant effect (electronic supplementary material, table S1).

## 3. Results

**Figure 2.**
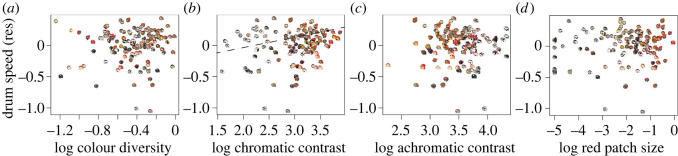
PGLS regressions between drum speed and plumage head coloration in woodpeckers. Dashed regression line represents the model for which significance was found. (*a*–*d*) represent the models between colour diversity, chromatic contrast, achromatic contrast and red area with residuals (res) of drum speed (after controlling for body mass). Data for all variables was log_10_ transformed. Use of modified illustrations was granted with permission by ©Lynx Edicions.

**Figure 3.**
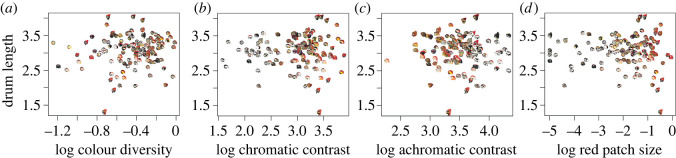
PGLS regressions between drum length and plumage head coloration in woodpeckers. (*a–d*) represent the models between colour diversity, chromatic contrast, achromatic contrast and red area with drum length. Data for all variables were log_10_ transformed. Use of modified illustrations was granted with permission by ©Lynx Edicions.

Using standard multivariate PGLS models, we tested for correlations between drum acoustics (speed and length) and metrics of head colour. With respect to drum speed (table 1), we uncovered a significant positive relationship with chromatic contrast (*p* = 0.011). This result indicates that species with faster drums are on average more likely to have greater colour boundary strength on their head plumage (figure 2*b*). By contrast, we found no relationship between drum speed and colour diversity (*p* = 0.636, figure 2*a*), size of the red area on the head (*p* = 0.148, figure 2*d*), and achromatic contrast (*p* = 0.939, figure 2*c*). For drum length (table 2), we found no evidence of a relationship with colour diversity (*p* = 0.297, figure 3*a*), chromatic contrast (*p* = 0.586, figure 3*b*), achromatic contrast (*p* = 0.895, figure 3*c*), or size of the red area in the head (*p* = 0.324, figure 3*d*). In this same vein, multivariate PGLSs showed no effect of habitat (open versus closed) on species variation in either drum speed or length (*p* = 0.960, table 1; *p* = 0.887, table 2) or measures of colour diversity (*p* = 0.956, electronic supplementary material, table S2), chromatic contrast (*p* = 0.485, electronic supplementary material, table S3), achromatic contrast (*p* = 0.267, electronic supplementary material, table S4) and size of red area of the head (*p* = 0.908, electronic supplementary material, table S5).

**Table 1.** PGLS model of colour traits and habitat predicting drum speed residuals from a linear regression with body mass (see text). Lambda: 0.877, *F*-statistic: 1.369, 5 and 126 d.f., *p*-value: 0.2405. Italics indicate statistically significant relationship.

term	estimate	s.e.	*t*-value	*p*-value
(intercept)	−0.5970	0.3303	−1.8076	0.0731
colour diversity	0.0398	0.0840	0.4743	0.6361
*chromatic contrast*	*0*.*1535*	*0*.*0596*	*2*.*5764*	*0*.*0111*
achromatic contrast	0.0051	0.0674	0.0759	0.9396
red patch size	−0.0405	0.0278	−1.4541	0.1484
habitat	0.0021	0.0420	0.0492	0.9608

**Table 2.** PGLS model of colour traits and habitat predicting drum length. Lambda: 0.715, *F*-statistic: 0.368, 5 and 126 d.f., *p*-value: 0.8697.

term	estimate	s.e.	*t*-value	*p*-value
(intercept)	2.7748	0.5433	5.1071	0.0000
colour diversity	0.1495	0.1429	1.0459	0.2976
chromatic contrast	0.0567	0.1040	0.5454	0.5864
achromatic contrast	0.0150	0.1137	0.1315	0.8956
red patch size	−0.0465	0.0470	−0.9896	0.3243
habitat	0.0102	0.0719	0.1413	0.8879

We corroborated our findings by running a second set of PGLS models, but this time we used the behavioural traits (drum speed and length) and the habitat type as predictors of the colour traits. One cannot necessarily assume that one signal trait is the actual predictor of the other because phenotypic traits often compromise each other mutually [76], and so this approach allows us to determine the robustness of our findings related to correlated evolution of display components across modalities. Our results here largely corroborated our previous findings, in that we again found a positive and significant relationship between chromatic contrast and drum speed (electronic supplementary material, table S3, *p* = 0.026), but no relationship with the drum length (*p* = 0.493). This analysis also showed that there is no significant correlation between drum length and any of the plumage metrics (*p* > 0.05, electronic supplementary material, tables S2–S5). Drum speed was not related to any other plumage metric (*p* > 0.05, electronic supplementary material, tables S2, S4, S5).

## 4. Discussion

Our study uncovers evidence of correlated evolution between woodpecker drumming behaviour and head colour elaboration, but we find that this correlation only exists with respect to certain components of these two signal modalities. Specifically, interspecific variation in the chromatic contrast of the woodpecker head plumage positively predicts the speed of the woodpecker drum, but the latter variable does not relate to species differences in head colour diversity, size of the head red patch or black and white contrast. Furthermore, we find no relationships between interspecific variation in drum length and measures of head colour, nor do we find a relationship between species differences in head colour or drumming behaviour with respect to habitat. Taken together, our results support the idea that woodpecker drum and head colour coevolve, though only with respect to certain elements of these signals.

We hypothesize that the positively correlated evolution we find between drum speed and head plumage chromatic contrast is a signature of display amplification [77]. Studies that explore this phenomenon propose that amplification acts to increase the probability of detection and/or discrimination of a second signal, essentially making it more conspicuous. For instance, in *Schizocosa* wolf spiders, the ornamentation found on the forelegs of males of certain species has been proposed to increase the effectiveness of the leg waving display in eliciting female receptivity [78]. In the case of woodpeckers, a highly conspicuous plumage (with high chromaticity) being added to the fast-moving head might act to increase the receiver's attention. Another possibility is that positively correlated evolution between drum speed and colour helps enhance signal efficacy. Often, communication can be hindered by the trade-off between signal detection and localization, given that the further a signal travels, the harder it can be to determine its source [79]. Multimodal signals can offer a solution to this issue because they can contain different elements that separately solve each of these problems [80]. In Tungara frogs, for example, females use visual cues supplied by the inflation of the vocal sac to help localize males who are calling in a large chorus [81]. For woodpeckers, faster drums may be harder to localize due to the effects of reverberation and signal degradation [82], and thus more visually conspicuous head plumage might help ameliorate this effect.

There are certainly additional explanations for the positive relationship between drum speed and head colour contrast. One intriguing possibility, for instance, is that correlated evolution of these signals forms the basis of a deceptive strategy to reduce detectability by predators. Woodpeckers drum frequently during the breeding season, which similar to other displaying behaviours can make them more susceptible to predation [35]. In some species, combining high-speed movement (like drumming) with a visual signal that has high contrast (like the plumage phenotype of the most conspicuous woodpecker heads) can create a visual illusion that makes it difficult for predators to track and capture an individual [83,84]. Although there are several ways such ‘optical illusions' can manifest, this idea is highly speculative and therefore requires further research. Nonetheless, our point is that contexts like this one may create scenarios in which we expect selection to favour positively correlated evolution between drum speed and head colour.

Of course, we also find that drum length is not related to any component of head colour. This non-significant result must be interpreted with caution, but it stands in notable contrast to our results in the context of drum speed. We suspect that differential relationships between head colour and drum speed and length are rooted in the function of the latter two display components. Drum length, for example, is probably tied closely to intraspecific competition for access to breeding territories and/or mates. Field studies show that longer drums elicit a more aggressive response from resident birds [41], while comparative work reveals that indices of the strength of intrasexual selection (size dimorphism) positively predict species variation in drum length [40]. In this way, if head plumage colour plays a minimal role in intraspecific sexual competition, then selection may not tie the elaboration of head colour to elements of the drum that do. Drum speed, on the other hand, may be more closely associated with male–female interactions. This idea has not been directly tested *per se*, but there are several studies that suggest that display speed is used to mediate elements of courtship in a wide range of taxa [42,85,86]. In a similar vein, plumage colour is also often used to help mediate courtship interactions [21,87], and thus the evolutionary elaboration of these two displays traits may become intertwined (see above for a mechanistic explanation).

Notably, common plumage patterns, such as the achromatic contrast, do not seem to be related to either drumming traits. We suspect that this is because black and white colour patterning in woodpeckers is more likely to be linked to predator avoidance, than it is to sexual communication [88]. Likewise, we found that our colour diversity metric was not associated with any of our drumming traits, indicating that species differences in the richness, distinctiveness and proportion of colours on the woodpecker's head is not evolutionarily coupled with drumming. Lastly, we do not find any relationship between the drumming metrics and the size of the red patch on the head of male woodpeckers. Again, these findings must be interpreted carefully, but one possibility is that these results arise from the fact that strong colour edge contrast is the most salient functional feature of head colour. This idea is based on the notion that chromatic contrast represents the combined differences of hue and chroma between adjacent patches, such that strong edge contrast becomes the primary means by which receiver attention is drawn and retained [89,90].

More broadly, our results show at least some consistence with other studies that do not find evidence of correlated evolution between the elaboration of different signal modalities [27,28,91]. It is largely unclear why some groups of species (e.g. taxonomic families) show such relationships, whereas others do not. Our results suggest that one explanation is that researchers are not necessarily looking at the right aspects of display elaboration (i.e. colour metric or behavioural metric). Another possibility, however, is that coevolutionary relationships between display traits appear (or not) as a function of display elaboration itself. In other words, display traits may only be related to each other (positively or negatively) when the display in question has surpassed a threshold of elaboration. If so, then we may find no evidence of correlated evolution between display elements simply because these taxa do not maintain signals that are *elaborate enough* to influence how other signals might evolve. Few studies have examined this idea in detail, but one way to do so might be to test for relationships between display variables at lower and upper boundaries of elaboration levels. This approach could reveal biological processes that more traditional ordinary least squares (OLS) or PGLS models cannot uncover [92,93]. However, novel phylogenetic comparative methods must be developed that allow the statistical testing of such ideas.

Despite many of the arguments above, we cannot entirely rule out how other factors might influence our results. Habitat, for example, can play a major role in shaping signal design [5,23]. We looked at this possibility by assessing whether life in an open or closed habitat predicts species differences in drumming behaviour, as well as head colour. We found no evidence of such an effect, which is consistent with the hypothesis that habitat openness does not strongly shape the evolution of drum behaviour or head plumage colour. For drumming, this could be expected since it is an atonal non-frequency-modulated signal, which might be less susceptible to the effects of the acoustic adaptation hypothesis proposed by sensory drive [94]. In the case of plumage coloration, previous studies report that woodpeckers' plumage is under strong selection for interspecific mimicry associated with geographical overlap, while being influenced by climatic variables such as temperature and precipitation [46]. These birds' plumage might therefore be affected by other selective forces that are not captured by our assessment of open versus closed habitat, even if this approach to environmental quantification worked well in other studies that assess effects of sensory drive [28,63]. All to say, we suspect that factors related to habitat probably do not explain our current results about the relationship between drumming behaviour and head colour.

## 5. Conclusion

It is currently unclear why some groups of species (e.g. taxonomic families) show evidence of correlated evolution between components of their social displays, whereas other groups of species do not. We use a variety of statistical approaches to assess the relationship between species differences in woodpecker drumming behaviour and head colour. We find that the relationship between such variables differs markedly depending on signal metric under consideration. Our results therefore suggest that multimodal signals evolve through highly complex processes, in which different elements of the signal shape how others arise and change through time in a manner that depends on their immediate (and probably past) functions. Future work should similarly consider this complexity when assessing correlated evolution among signal components, as our understanding of the origins of multimodal signalling system is probably far more complex than we currently appreciate.

## Data Availability

The data are provided in electronic supplementary material [95].
